# A real-time quantitative polymerase chain reaction for the specific detection of *Hammondia hammondi* and its differentiation from *Toxoplasma gondii*

**DOI:** 10.1186/s13071-020-04571-8

**Published:** 2021-01-25

**Authors:** Gereon Schares, Majda Globokar Vrhovec, Mareen Tuschy, Maike Joeres, Andrea Bärwald, Bretislav Koudela, Jitender P. Dubey, Pavlo Maksimov, Franz J. Conraths

**Affiliations:** 1 National Reference Laboratory for Toxoplasmosis, Institute of Epidemiology, Friedrich-Loeffler-Institut, Federal Research Institute for Animal Health, Südufer 10, 17493 Greifswald—Insel Riems, Germany; 2 IDEXX Laboratories, Humboldtstraße. 2, Kornwestheim, 70806 Germany; 3 Central European Institute of Technology (CEITEC), University of Veterinary and Pharmaceutical Sciences, Palackého tř. 1946/1, Brno, 612 42 Czech Republic; 4 Faculty of Veterinary Medicine, University of Veterinary and Pharmaceutical Sciences, Palackého tř. 1946/1, Brno, 612 42 Czech Republic; 5 Animal Parasitic Diseases Laboratory, Beltsville Agricultural Research Center, Agriculture Research Service, United States Department of Agriculture, Building 1001, Beltsville, MD 20705-2350 USA

**Keywords:** *Hammondia hammondi*, Oocyst, Faecal examination, TaqMan polymerase chain reaction, Quantitative polymerase chain reaction

## Abstract

**Introduction:**

*Hammondia hammondi* and *Toxoplasma gondii* are closely related protozoan parasites, but only *T. gondii* is zoonotic. Both species use felids as definitive hosts and cannot be differentiated by oocyst morphology. In *T. gondii*, a 529-base pair (bp) repetitive element (TgREP-529) is of utmost diagnostic importance for polymerase chain reaction (PCR) diagnostic tests. We identified a similar repetitive region in the *H. hammondi* genome (HhamREP-529).

**Methods:**

Based on reported sequences, primers and probes were selected* in silico* and optimal primer probe combinations were explored, also by including previously published primers. The analytical sensitivity was tested using serial dilutions of oocyst DNA. For testing analytical specificity, DNA isolated from several related species was used as controls. The newly established TaqMan PCR (Hham-qPCR1) was applied to tissues collected from *H. hammondi*-infected gamma-interferon gene knockout (GKO) mice at varying time points post-infection.

**Results:**

Ten forward and six reverse primers were tested in varying combinations. Four potentially suitable dual-labelled probes were selected. One set based on the primer pair (Hham275F, Hham81R) and the probe (Hham222P) yielded optimal results. In addition to excellent analytic specificity, the assay revealed an analytical sensitivity of genome equivalents of less than one oocyst. Investigation of the tissue distribution in GKO mice revealed the presence of parasite DNA in all examined organs, but to a varying extent, suggesting 100- to 10,000-fold differences in parasitic loads between tissues in the chronic state of infection, 42 days post-infection.

**Discussion:**

The use of the 529-bp repeat of *H. hammondi* is suitable for establishing a quantitative real-time PCR assay, because this repeat probably exists about 200 times in the genome of a single organism, like its counterpart in *T. gondii*. Although there were enough sequence data available, only a few of the primers predicted *in silico* revealed sufficient amplification; the identification of a suitable probe was also difficult. This is in accord with our previous observations on considerable variability in the 529-bp repetitive element of *H. hammondi*.

**Conclusions:**

The *H. hammondi* real-time PCR represents an important novel diagnostic tool for epidemiological and cell biological studies on *H. hammondi* and related parasites. 
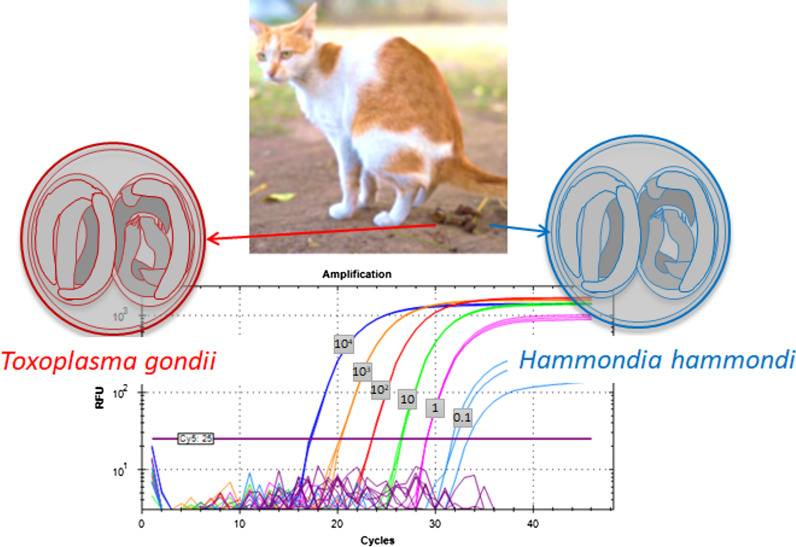

## Background

*Hammondia hammondi*, a coccidian parasite, is closely related to the zoonotic protozoan *Toxoplasma gondii* [1, 2]. Similar to *T. gondii*, *H. hammondi* uses felids such as the domestic cat as its definitive hosts [3]. Oocysts of *T. gondii* and *H. hammondi* are morphologically indistinguishable [3, 4]. Thus, faecal examinations solely based on microscopy cannot be used to estimate the prevalence of *T. gondii* or *H. hammondi* oocysts in feline hosts [5–7].

Laboratory mice, rats, hamsters, guinea pigs, wild rodents, rabbits, goats and dogs are susceptible to infection with *H. hammondi* oocysts [3, 6, 8–14]. While monkeys [15] are also experimental intermediate hosts of *H. hammondi*, no *H. hammondi* infection has so far been demonstrated in humans. No avian species, including chickens [16], quails [13] and pigeons [10, 13], could be infected with *H. hammondi*, which suggests avians are not its intermediate hosts [7]. In contrast to *T. gondii*, *H. hammondi* infections are basically asymptomatic in both definitive and intermediate hosts [3].

While several polymerase chain reactions (PCRs) are available for the diagnosis of *T. gondii* infections, only a few diagnostic PCRs for *H. hammondi* have been reported so far. Those for *T. gondii* are based on a variety of targets, including the B1 gene [17], the internal transcribed spacer (ITS)-1 region of ribosomal DNA (rDNA) [18] and a 529-base pair (bp) repetitive element, also known as TgREP-529 (GenBank AF146527) [19, 20]. The ones reported for *H. hammondi* are end-point PCRs, which use as targets ITS-1 rDNA [21] or a 529-bp repeat, which is similar to the *T. gondii* repetitive element and is designated here as HhamREP-529 [6]. To the best of our knowledge, no real-time PCR for the diagnosis of *H. hammondi* infections has so far been published. The availability of a real-time PCR will allow simplification of *H. hammondi* prevalence estimates. It will also allow the detection and quantification of oocysts of this parasite in feline faeces and in tissues of natural and experimental intermediate hosts.

Since TgREP-529 of *T. gondii* exists up to about 200 times in the genome of a single organism, as recently shown by third-generation sequencing [22], and sequence information on a homologue of this repetitive element in *H. hammondi* was readily available from prior studies [6], we decided to establish a HhamREP-529-based real-time PCR for *H. hammondi*. Unexpectedly, we faced several problems when we tried to establish a sensitive primer and probe combination, probably due to sequence variations between individual repeats, as observed earlier [6].

## Methods

### Parasite strains to generate reference DNA

Field isolates of *H. hammondi* oocysts were made available by IDEXX Laboratories, Ludwigsburg, Germany, and the University of Veterinary and Pharmaceutical Sciences, Brno, Czech Republic, in the frame of projects that are aimed at obtaining European *T. gondii* isolates (Additional file 1: Table S1). Faecal samples were tested by flotation and examined microscopically as described previously [7]. If *T. gondii*-like oocysts were detected, purified oocysts were genotyped at the species level by PCR using DNA extracted from oocysts. Occasionally, gamma interferon gene knockout (GKO) mice [C.129S7(B6)-Ifngtm1Ts/J; The Jackson Laboratory, Bar Harbor, ME] were in addition inoculated with sporulated oocysts via oral gavage; parasites were recovered as described previously [23] and genotyped. One *H. hammondi* oocyst isolate from Iran had been kindly provided by Dr. Morteza Hosseininejad, Faculty of Veterinary Medicine, Shahr-e Kord, Iran, in 2010. Oocysts of the isolate H.H.34 were from the USA, and whole genome sequences (GenBank: AHJH01004981.1) were recently obtained for these. H.H.34 oocysts were used to infect GKO mice to obtain tissue stages for DNA extraction, and DNA was also extracted from the oocysts (Additional file 1: Table S1; Additional file 2: Table S2).

To confirm the specificity of the Hham-qPCR1, we used DNA from three *T. gondii* strains (RH, ME49, NED), *Neospora caninum* (Nc-1), *Besnoitia besnoiti* (Evora), *Hammondia heydorni*, *Eimeria bovis*, *Cystoisospora felis*, *Cystoisospora rivolta*, *Cystoisospora burrowsi*, *Cystoisospora canis*, *Cryptosporidium parvum*, *Sarcocystis hirsuta*, *Sarcocystis bovifelis*, *Sarcocystis hominis*, *Sarcocystis cruzi*, *Giardia* spp. and *Tritrichomonas foetus*, in addition to DNA isolated from *Hammondia hammondi*. *T. gondii*, *N. caninum* and *B. besnoiti* were obtained by* in vitro* cultivation of the parasites, which yielded reference samples consisting of 100 ng/µl concentrated pure DNA. For the remaining oocyst reference DNA samples, DNA was extracted from field isolates (*H. heydorni*, *Cystoisospora* spp., *Sarcocystis* spp.; 10^4^–10^5^ oocysts or sporocysts) yielding 100 µl DNA for each parasite. *Eimeria bovis* oocysts were kindly provided by Dr. Christian Bauer, Institute for Parasitology, Giessen, Germany. Oocysts of *C. parvum* (Germany) were kindly provided by Prof. Dr. A. Daugschies, Institute of Parasitology Leipzig, Germany. *Giardia* spp. and *T. foetus* DNA were kindly provided by Dr. Christian Klotz, Robert Koch-Institut, Berlin, Germany and Dr. Klaus Henning, Friedrich-Loeffler-Institut, Jena, Germany.

### Experimental infections of mice

GKO mice (*n* = 28) were orally inoculated with different isolates and varying doses, depending on the availability of field isolates, in experiments to isolate *T. gondii* or to trigger the permanent growth of *H. hammondi* in mice and eventually in cell culture (Additional file 2: Table S2). Sporulated oocysts were counted using a Neubauer chamber and mice infected via oral gavage as described [23].

### DNA extraction

Oocysts were isolated from faeces using combined sedimentation and flotation by adding 13 ml concentrated sucrose (specific gravity of 1.3) to 1 ml faecal sediment as described previously [24]. Floating oocysts were collected, washed three times by centrifugation (1100× *g*, 7 min, without brake) and a five- to tenfold volume of phosphate-buffered saline. DNA was extracted from the final pellet using a phenol/chloroform purification method as previously described [23] or using the NucleoSpin Soil kit (Macherey and Nagel, Düren, Germany) following the manufacturer’s recommendations.

Tissue samples (25 mg) were extracted by using the NucleoSpin Tissue kit (Macherey and Nagel) following the manufacturer’s recommendations. Because it was not the aim of the present study to estimate parasite DNA concentration per infected cell but per type of tissue, DNA samples from tissues were not normalized but used as extracted.

### Conventional endpoint PCRs

To confirm the presence of *H. hammondi* in oocyst samples, a previously published end-point PCR was applied as described to examine oocyst DNA [4].

To test DNA extracted from oocyst samples for coccidian DNA, a PCR was performed using the common apicomplexan small subunit ribosomal DNA (SSU-rDNA) primers COC-1 and COC-2 [25]. Primers were used at a final concentration of 0.5 mM and deoxynucleotide triphosphates at a final concentration of 250 mM each (Stratec Molecular, Berlin, Germany). Taq polymerase (Stratec Molecular) was used at 1U/25 μl with the buffer system supplied with the enzyme. The PCR cycling conditions were the same as previously reported [26]. To confirm the presence of *Sarcocystis* spp., primers SarcoFint and SarcoRint specific for *Sarcocystis* spp. SSU-rDNA were used as previously described [27]. To test for *Giardia* spp. DNA, the published primers AS1 GiardiaF and AS2 GiardiaR [28] were used, and for *T. foetus* DNA, we used the primer pair TFR1/TFR2 [29]. All reagents, except for primers, were the same as described above. They were used in the same concentrations as described for the coccidian PCR. The PCR cycling conditions were 94 °C for 5 min, followed by 35 cycles of 60 °C for 1 min, 72 °C for 1 min and 94 °C for 1 min. The PCR ended with a final extension at 72 °C for 10 min.

### Identification of primer pairs suitable for amplifying *H. hammondi* DNA

To identify optimal primer pairs, a SybrGreen real-time PCR was performed using DNA (D9494) from a *H. hammondi* isolate (VB919008), diluted to resemble the DNA of approximately 5 oocysts/µl DNA. All possible combinations of forward and reverse primers (Additional file 3: Table S3) were checked in a SybrGreen real-time PCR using the iTaq Universal SYBR Green Super mix (Bio-Rad Laboratories, Munich, Germany) to test the *H. hammondi* template in comparison to a no-template control (DNA-grade water). Each primer pair was evaluated twice in two independent SybrGreen real-time PCRs and the results were recorded as mean Δ threshold cycle (Ct)_pos-neg_, i.e. the ΔCt between results (Ct values) for the *H. hammondi* template and the no-template control (Additional file 4: Table S4).

### Novel real-time PCR to detect *H. hammondi* DNA

The quantitative TaqMan real-time PCR was performed as described previously [26], including the integration of an internal control (IC) system [26, 30]. The novel real-time PCR employing the optimal primer-probe combination was designated Hham-qPCR1. To monitor inhibition in real-time PCR, a heterologous plasmid DNA resembling the enhanced green fluorescent protein (EGFP) gene [30] was added to the reaction mix including the primers EGFP1-F, EGFP2-R and the probe EGFP1 [26]. A 712-bp fragment of the EGFP gene was amplified as the EGFP template and cloned into the pGEMTeasy standard cloning vector (Promega, Walldorf, Germany) in reverse orientation to obtain IC-2 DNA (pGEM-EGFP2-rev). The amount of IC-2 DNA added to each reaction was adjusted in such a way that it resulted in a Ct value of about 32 in the real-time PCR. Reactions were performed in a final volume of 20 μl using a commercial master mix (PerfeCTa MultiPlex qPCR ToughMix; Quantabio, VWR International, Darmstadt, Germany) and a CFX96 instrument (Bio-Rad Laboratories). Primers and probes (Additional file 3: Table S3) were purchased from MWG-Biotech (Ebersberg, Germany).

Standard concentrations for primers (500 nM; Additional file 3: Table S3) and probes (100 nM for *H. hammondi* probes, Cy5 labelled; Additional file 3: Table S3; EGFP1, HEX labelled [26]) were applied. These standard concentrations were also applied to the finally selected primer-probe combination (Hham275F, Hham81R, Hham222P). The cycling conditions in the Hham-qPCR1 were 95.0 °C (5 min, initial denaturation), followed by 45 cycles, during which the samples were first incubated at 95.0 °C for 10 s and then at 60.0 °C for 30 s. After each cycle, light emission by the fluorophore was measured. Real-time PCR results were analysed using the CFX manager software version 1.6 (Bio-Rad Laboratories).

### Sensitivity and specificity of Hham-qPCR1

The analytical sensitivity was determined using a *H. hammondi* oocyst isolate with a high sporulation rate (P18/2900; sporulation rate 84%). Tenfold dilutions of three DNA samples isolated from 10^5^ oocysts were tested by the Hham-qPCR1 in twofold repetition including the EGFP IC.

To confirm that the novel PCR recognized a broad range of *H. hammondi* specimens, oocyst isolates from Germany and other countries were tested by Hham-qPCR1. These DNA samples included oocyst DNA isolated from 19 *H. hammondi* samples of different origin from Germany, four samples from Denmark, two from Austria, and one each from France, the Czech Republic and the USA, i.e. *n* = 28 samples in total (Additional file 1: Table S1). The presence of *H. hammondi* had been confirmed by our previously published end-point PCR [6]. All these samples had tested negative in a Tg-qPCR [31, 32].

The analytical specificity was confirmed using DNA samples of parasites closely related to *H. hammondi*, which are mentioned in the “Parasite strains to generate reference DNA” section. The presence of DNA in these samples was confirmed by the SSU-rDNA end-point PCR as detailed in the “Conventional endpoint PCRs” section.

### Statistical analysis

Calculations of medians and SDs were performed using EXCEL 2019, Microsoft Office (Microsoft, Seattle, WA).

To determine the relatedness of real-time PCR results and oocyst concentrations in samples, linear regression was performed using the lm command in R version 3.5.3 (R Core Team 2017). For the analysis, oocyst concentrations (number of oocysts per millilitre) were log 10 transformed.

Figures were assembled using R versions 3.5.3 or 4.0.0 (packages ggplot2, reshape and scales). Box plots displaying real-time PCR data of infected mice show the median, 25th and 75th percentiles, 1.5 interquartile ranges and outliers; PCR results were considered negative at a cut-off Ct value of 45.

## Results

### Location of primers

**Fig. 1:**
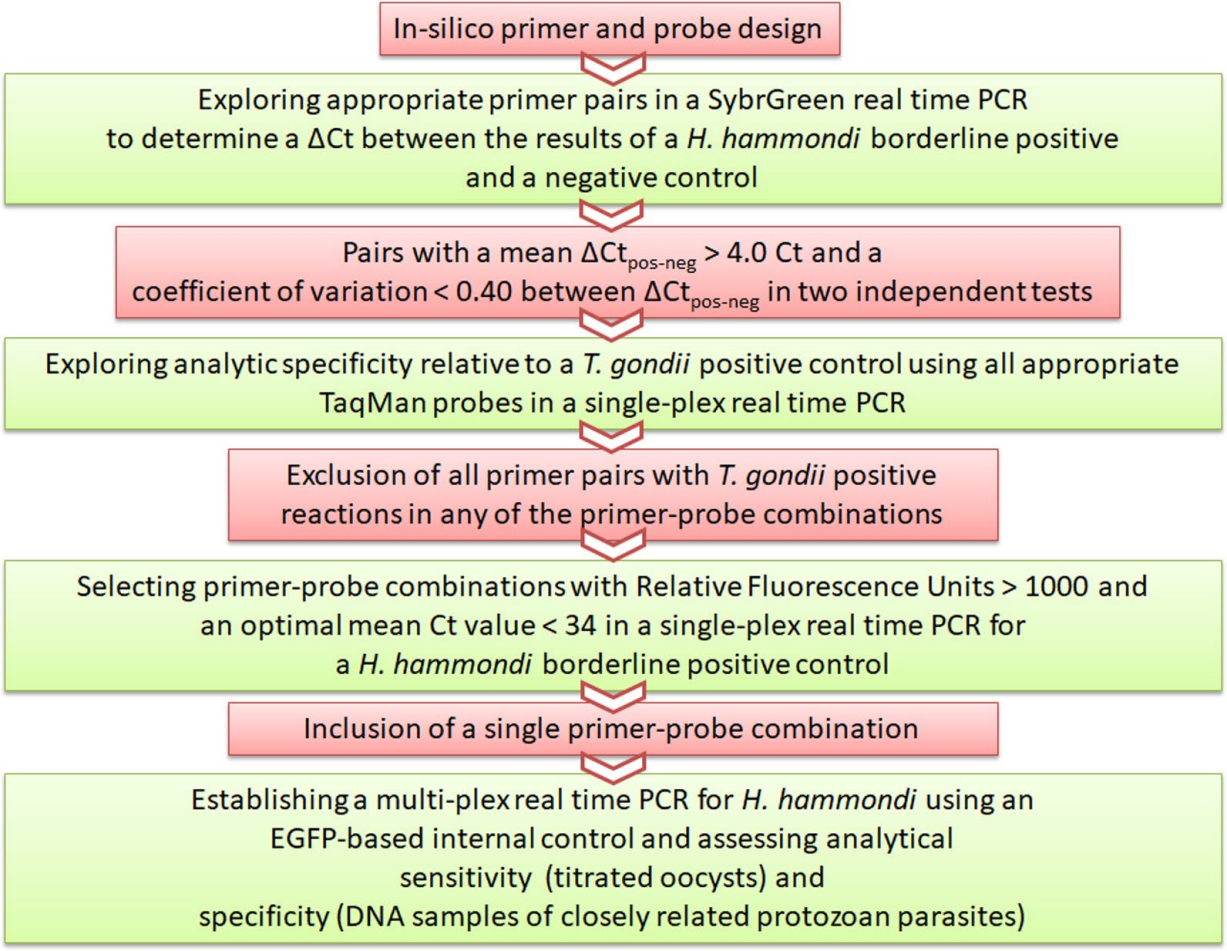
Workflow for establishing a quantitative real-time polymerase chain reaction for *Hammondia hammondi* (Hham–qPCR1) in oocysts and intermediate host tissues using a sensitive and specific primer-probe combination and an enhanced green fluorescent protein (*EGFP*)-based internal control (IC)

Potentially suitable primers and probes (Additional file 3: Table S3) for two 5’-nuclease quantitative real-time PCR assays were selected by* in silico* methods (Fig. 1). To this end, published sequences (*n* = 9) of HhamREP-529 were downloaded from GenBank (KC223619, JX477424, EU493279, EU493280, EU493281, EU493282, EU493283, EU493284, EU493285) and aligned with Geneious 10.0.9 (Multiple Geneious Alignment, Global alignment with free end gaps, Gap open penalty 12, Gap extension penalty 3, refinement iterations 2). Primers were selected to amplify a fragment with a maximum size of about 150–160 bp.

### Identification of optimal primer pairs to amplify *H. hammondi* DNA

**Fig. 2:**
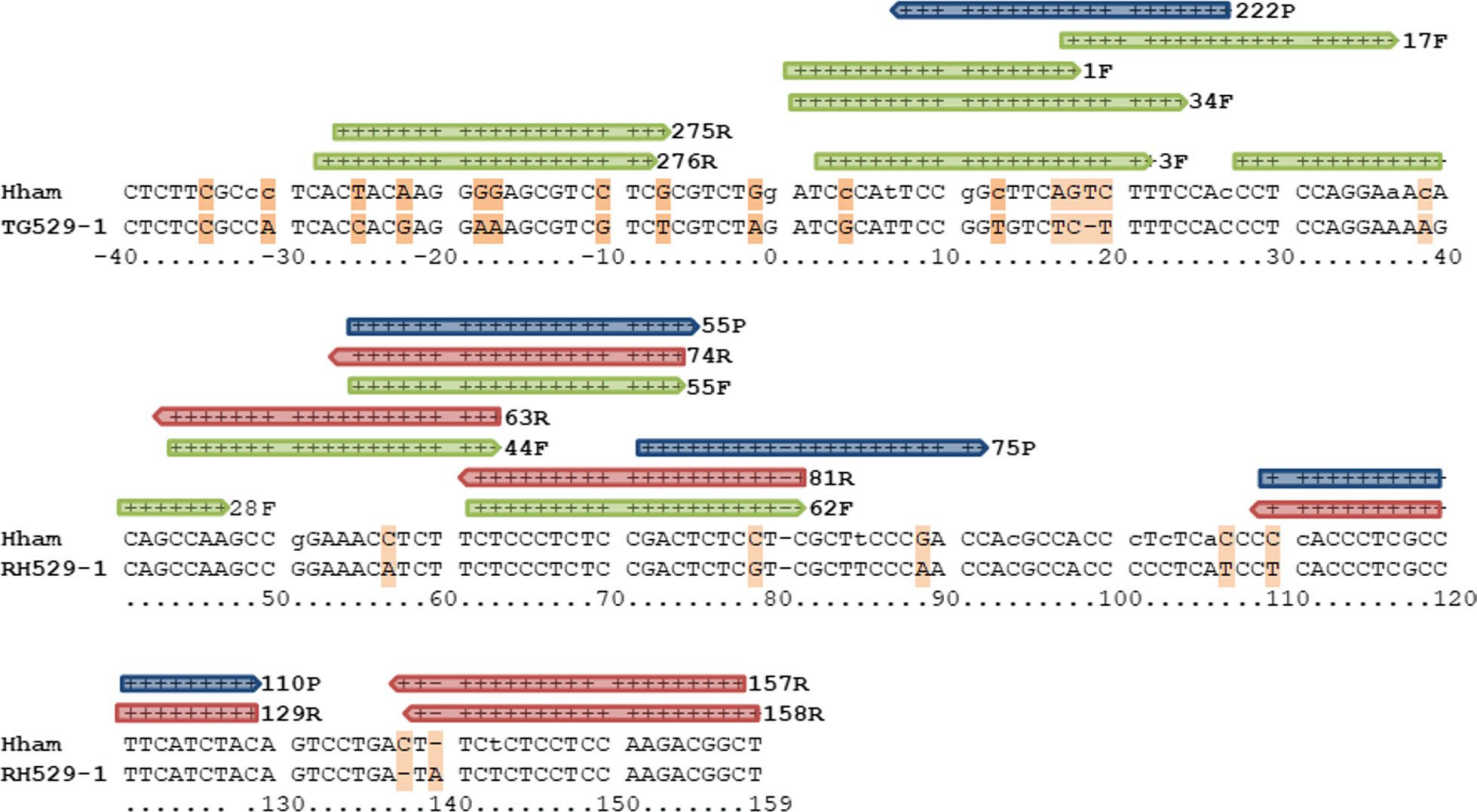
Locations of primers and probes on a fragment of the 529-base pair (bp) repeat sequence of *Hammondia hammondi* and *Toxoplasma gondii*. Consensus sequences of *H. hammondi* have been deposited in GenBank (KC223619, JX477424, EU493279, EU493280, EU493281, EU493282, EU493283, EU493284, EU493285); the RH529-1 sequence has been previously published [19]. Further details are provided in Additional file 3: Table S3. There are several sequence variants of *H. hammondi*;* lowercase letters* indicate polymorphic sites.* Blue* indicates a probe,* green* indicates a forward primer,* red* indicates a reverse primer, *orange* indicates a sequence difference, *arrow-headed end* indicates a 5′-end

In total, 39 primer pairs were evaluated with a SybrGreen assay and *H. hammondi* reference DNA D9494. Out of 51 primer pairs tested,* n* = 22 revealed ΔCt_pos-neg_ values of > 4. Five of these 22 pairs revealed a large difference between two independent tests and in 17, the coefficient of variation of ΔCt_pos-neg_ (Additional file 4: Table S4) was less than 0.4. These 17 primer pairs were further validated with up to four of the probes Hham55P, Hham75P, Hham110P or Hham222P (Additional file 3: Table S3) depending on the location of primers and probes (Fig. 2). Eleven of the 28 primer-probe combinations were excluded due to cross-reaction with *T. gondii* DNA (1000 ng). Four of the remaining primer-probe combinations showed mean relative fluorescence unit values > 1000 and mean Ct values of 33.6 to 35.3 in from three to five independent tests. The finally selected primer-probe combination consisted of the primers Hham275F and Hham81R and the probe Hham222P (Fig. 2; Additional file 3: Table S3). The analytical sensitivity and specificity were assessed for this primer-probe combination in a multiplex real-time PCR, in which EGFP-specific reagents were included as an IC to assess PCR inhibition, in addition to *H. hammondi*-specific reagents. This assay was designated as the Hham-qPCR1.

### Analytical sensitivity and efficiency of the Hham-qPCR1

**Fig. 3a, b:**
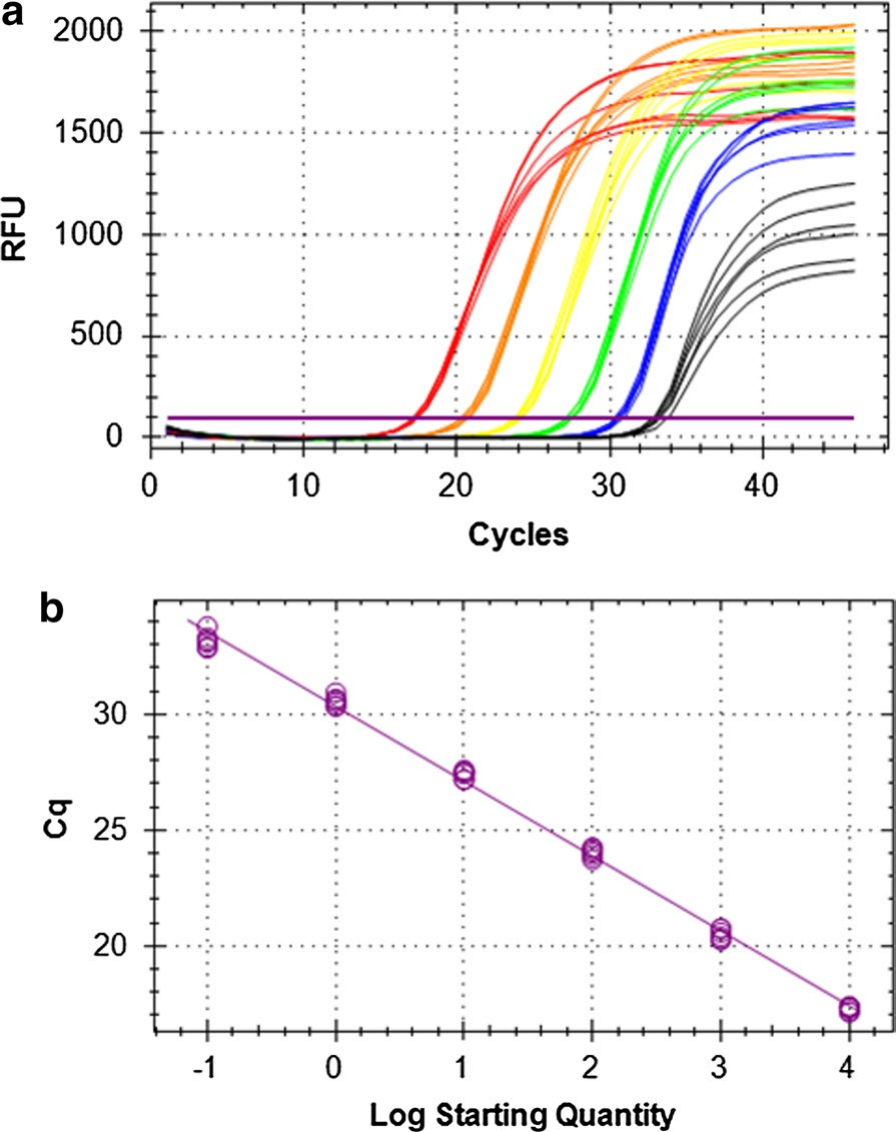
Characteristics of the Hham-qPCR1 with an EGFP IC. **a** DNA equivalents of 0.1 *Hammondia hammondi* oocyst reacted positively in the PCR [colours represent different DNA equivalents: 10^4^ oocysts (*red*); 10^3^ oocysts (*orange*); 10^2^ oocysts (*yellow*), 10 oocysts (*green*); 1 oocyst (*blue*); 0.1 oocyst (*black*)]. **b** The Hham-qPCR1 was characterized by a PCR efficiency (*E*) of 103.9%, and linear regression using DNA equivalents of 10^4^–10^−1^ oocysts yielded a coefficient of determination (*R*^2^) of 99.7%. *RFUs* Relative fluorescence units,* Cq* threshold cycle (Ct); for other abbreviations, see Fig. 1

The analytical sensitivity of the Hham-qPCR1 was determined using a German *H. hammondi* oocyst isolate, which showed a high sporulation rate. Tenfold dilutions of three DNA samples isolated separately from 10^5^ oocysts each were twice examined by the Hham-qPCR1 including the EGFP IC. A PCR efficiency (*E*) = 103.9%, a coefficient of determination (*R*^2^) = 99.7% and a slope of the regression line of 3.231 were recorded (Fig. 3).

To confirm that the Hham-qPCR1 recognizes a broader range of *H. hammondi* isolates, we analysed DNA of oocysts collected from cat faeces. These DNA samples included 19 samples from Germany, four from Denmark, two from Austria and one from France, the Czech Republic and the USA each, i.e. a total of 28 samples. In the new Hham-qPCR1 these samples yielded Ct values between 15.9 and 28.3, which is equivalent to the DNA of 10^2^–10^3^ oocysts per microlitre (Additional file 1: Table S1). There was a linear relationship between the logarithm (log 10) of the number of isolated oocysts per millilitre and the observed Ct values, which was characterized by an *R*^2^ of 17.5%.

### Analytical specificity

**Fig. 4:**
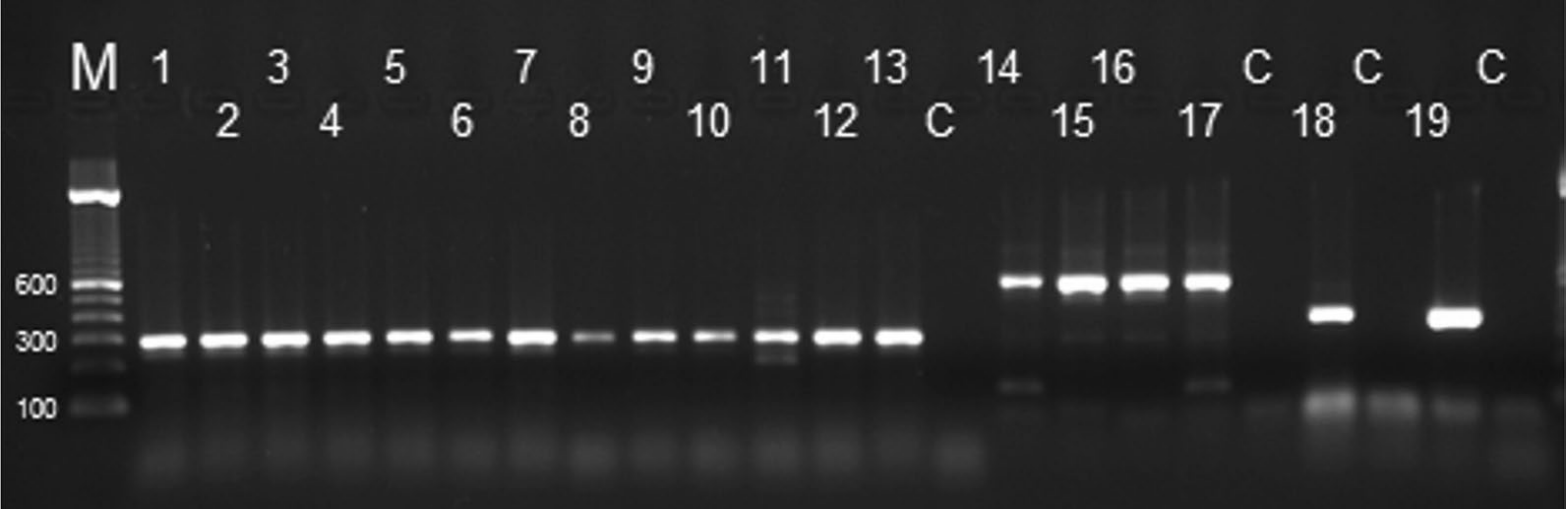
Confirmation that samples used to determine the analytical specificity of Hham-qPCR1 contained sufficient parasite DNA.* Lanes 1–19* *Hammondia hammondi*, *Toxoplasma gondii* (RH), *Toxoplasma gondii* (ME49), *Toxoplasma gondii* (NED), *Neospora caninum* (Nc-1), *Besnoitia besnoiti* (Evora), *Hammondia heydorni*, *Eimeria bovis*, *Cystoisospora felis*, *Cystoisospora rivolta*, *Cystoisospora burrowsi*, *Cystoisospora canis*, *Cystoisospora parvum*, *Sarcocystis hirsuta*, *Sarcocystis bovifelis*, *Sarcocystis hominis*, *Sarcocystis cruzi*, *Giardia* spp., *Tritrichomonas foetus.* DNA in* lanes 1–13* had been amplified with* Coccidia*-specific primers COC-1 and COC-2, in* lanes 14–17* with the *Sarcocystis* spp.-specific primers SarcoFint and SarcoRint, in* lane 18* by the *Giardia* spp.-specific primers RH11_RH4 and RH11_RH5, and in* lane 19* by the *Trichomonas* spp.-specific primers TRF1 and TRF2.* M* Marker (100 bp),* C* control (no template)

The analytical specificity of Hham-qPCR1 was assessed using DNA samples of parasites closely related to* H. hammondi*, i.e.* Toxoplasma gondii* (strains RH, ME49, NED),* N. caninum* (Nc-1),* B. besnoiti* (Evora),* H. heydorni*,* E. bovis*,* C. felis*,* C. rivolta*,* C. burrowsi*,* C. canis*,* C. parvum*,* S. hirsuta*,* S. bovifelis*,* S. hominis*,* S. cruzi* and DNA of parasites frequently observed in cat faeces like* Giardia* spp. and* Tritrichomonas foetus*. Presence of DNA in these samples was confirmed by end-point PCR (Fig. 4). In the Hham-qPCR1, no amplification with any of these DNA samples was observed.

### Examination of tissue stages of *H. hammondi*

**Fig. 5a, b:**
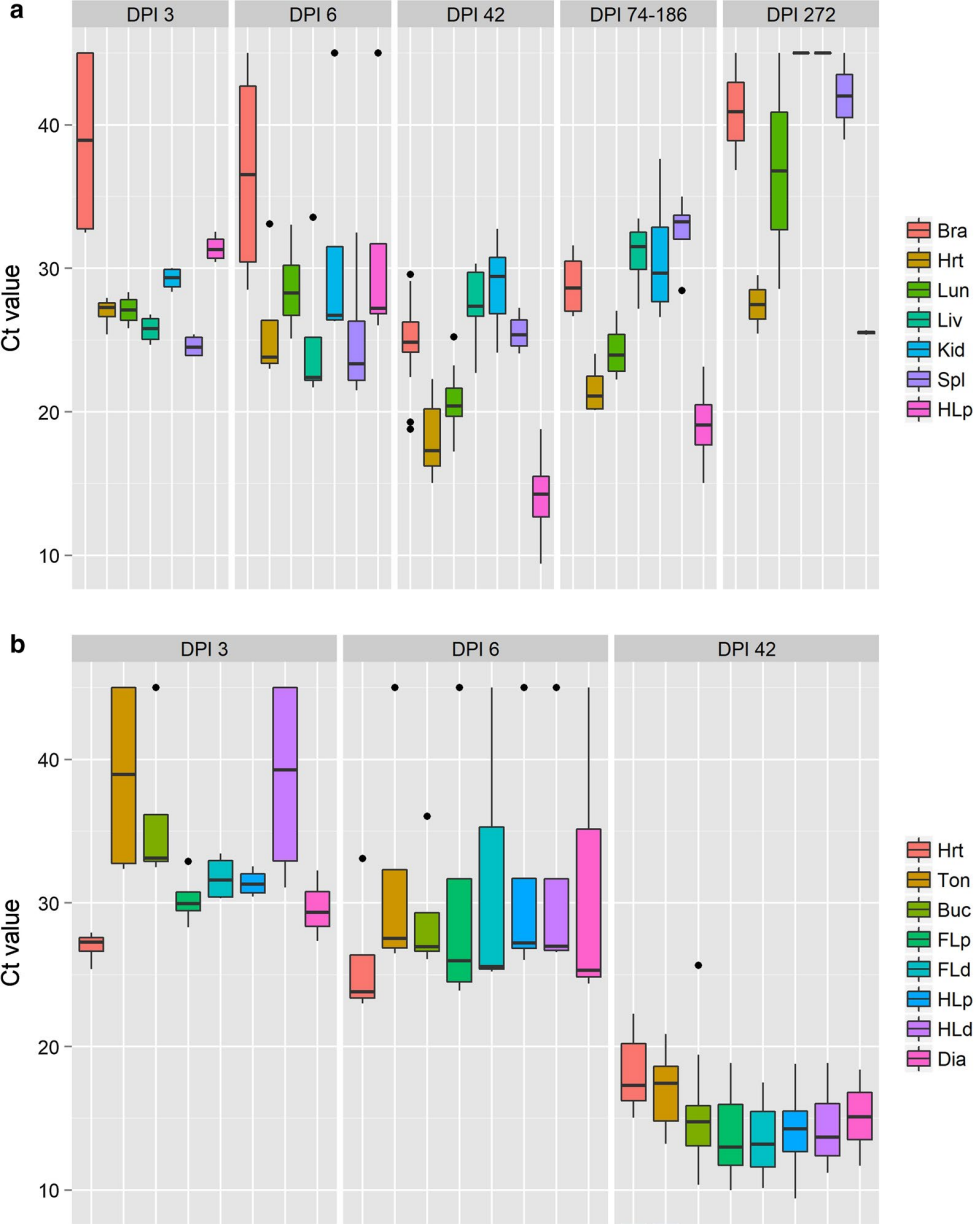
Hham-qPCR1 Ct values in various tissues of gamma interferon gene knockout mice inoculated with *Hammondia hammondi*. **a** For 28 mice necropsied 3, 6, 42, 74–186, or 272 days post-infection (*DPI*), seven tissues, brain (*Bra*), heart (*Hrt*), lung (*Lun*), liver (*Liv*), kidney (*Kid*), spleen (*Spl*) and the proximal hind limb musculature (*HLp*), were available. **b** For some of the mice, samples of the HLp, tongue (*Ton*), buccal musculature (*Buc*), proximal forelimb (*FLp*), distal forelimb (*FLd*), distal hind limb (*HLd*) and diaphragm (*Dia*) were also available and tested. Box plots show the median (*line*), 25th and 75th percentiles (*box*), 1.5 interquartile range (*whiskers*) and outliers (*dots*)

Tissues of 28 GKO mice infected with *H. hammondi* for varying time periods were available, from which DNA was extracted and tested by Hham-qPCR1 (Fig. 5a, b). From each of 28 mice, seven tissues (brain, heart, lung, liver, kidney, spleen and the proximal hind limb musculature) had been collected. In the first days post-infection (DPI; DPI 3, DPI 6), the median Ct values in Hham-qPCR1 ranged between 22.3 and 31.3 for organs including mostly muscle tissue (except for the distal hind limb and tongue, 39.2 or 38.9, respectively); by contrast, median Ct values in brain tissue ranged only from 36.5 to 39.9 (Fig. 5a). On DPI 42, skeletal muscle (i.e. proximal hind limb musculature) showed the lowest median Ct values (14.2) of all tissues in this comparison, followed by heart (17.3) and lung (20.4). Of the remaining organs, brain and spleen showed similar median Ct values (24.9 and 25.4). Liver and kidney also yielded Ct values in the same range (27.4 and 29.4). Later during infection (DPI 74–186 and 272), heart and skeletal muscle had the lowest median Ct values followed by lung, brain, kidney and spleen. However, Ct values at DPI 272 were considerably lower than during DPI 74–186 in mice (Fig. 5a).

Moreover, muscle tissues obtained from different locations on DPI 3, 6 and 42 were comparatively examined (Fig. 5b). On DPI 3 and 6, heart showed the lowest Ct values of all muscle tissues (median Ct 27.3 and 23.8), while heart and tongue had the highest Ct values on DPI 42 (Fig. 5b). The remaining muscle tissues showed very similar Ct values, i.e. medians ranged from 14.7 to 13.0. In all skeletal muscle tissues, the Ct values had dropped considerably between DPI 6 (Ct 27.0–25.3) and DPI 42 (Ct 10.2–13.3). In brain tissue, a similar decrease in Ct values was observed between DPI 6 and DPI 42. By contrast, for heart, a ΔCt of only 6.5 was observed between the median Ct values of DPI 6 and DPI 42 (Additional file 5: Table S5).

## Discussion

The present study reports on the development and characterization of a 5’-hydrolysis real-time PCR (TaqMan PCR) for the quantitative detection of *H. hammondi*. To the best of our knowledge, this is the first real-time PCR for the detection of *H. hammondi* DNA in feline faecal samples or intermediate host tissues.

There are numerous end-point and real-time PCRs for *T. gondii* that target an up to about 200-fold repeated element in the genome of the parasite (TgREP-529). We identified a similar 529-bp repeat in *H. hammondi* and established the Hham34F/Hham3R end-point PCR to diagnose *H. hammondi* infection several years ago [6]. This PCR was subsequently applied to test feline faecal samples [5, 7], rodent tissues [33] and used in cell biological studies to elucidate differences between the life cycles of *T. gondii* and *H. hammondi* [34]. Although the use of HhamREP-529 seemed to be ideal for establishing a quantitative real-time PCR, and enough sequence data were available, no real-time PCR had previously been established for *H. hammondi*.

We found that only a small set of the primers that we had predicted* in silico* revealed enough specificity and sensitivity for the amplification of *H. hammondi* DNA. The identification of a suitable probe was also more difficult than expected. In a previous study, we cloned and sequenced several PCR amplification products of HhamREP-529 and observed considerable sequence variation [6]. It seems possible that these sequence polymorphisms prevented many of the* in vitro*-selected primers and probes from binding sufficiently to DNA.

The HhamREP-529 and the TgREP-529 sequences are similar, but not identical (identity ranging from 93.7 to 97.5%; [6]), and the *H. hammondi* primers and probes that were finally selected showed a considerable number of mismatches with respect to the *T. gondii* sequence. To confirm the analytical specificity relative to *T. gondii*, we used DNA samples from the three major European and North American clonal lineages of *T. gondii*. In addition, we confirmed the specificity with a variety of DNA samples from parasite species or genera related to *H. hammondi* or *T. gondii*, including parasitic protozoa frequently observed in cat faeces like *Giardia* spp. or *T. foetus*. Our results indicate that Hham-qPCR1 has an excellent analytical specificity.

When we tested DNA samples of more than 20 *H. hammondi* isolates from Austria, Denmark, France, Germany and the USA (oocysts), the Czech Republic (oocysts, mouse tissue) and Iran (mouse tissue), there was no indication that our Hham-qPCR1 lacks sensitivity for particular isolates. Due to limited knowledge on the global population structure of *H. hammondi,* we cannot exclude, however, the possibility that yet unidentified lineages of *H. hammondi* exist, which may be genetically diverse. If there are further *H. hammondi* lineages, the possibility remains that our primer-probe combination could show limited or no binding to the DNA of such lineages. However, experience with *T. gondii*, which has several genetically diverse haplogroups, suggests that TgREP-529 is relatively conserved. In general, only a few failures of TgREP-529-based PCRs for the detection of *T. gondii* have been reported [35–40]. However, there are some cases in which other PCR assays either based on ITS-1 rDNA [41] or on B1 as a target [42] detected a considerable number of additional *T. gondii*-positive DNA samples compared to the number of positive results obtained by TgREP-525-based PCRs.

An earlier study observed only a few differences among various strains of the canonical clonal lineages regarding the number of TgREP-529 repeat units per organism [43], and a very recent study reports similar observations [22]. It has been discussed, but so far not investigated, to what extent sequence polymorphisms between the tested canonical clonal lineages contributed to these findings [43]. Future studies need to assess if there are differences in the number of HhamREP-529 repeats in strains of *H. hammondi*.

We validated the analytical sensitivity of the Hham-qPCR1 with oocyst DNA. Similar to the results of our previous study using an end-point PCR [6], the analytical sensitivity of the Hham-qPCR1 was equivalent to the DNA content of 0.1 oocyst. Considering that the 200 µg faeces, that we usually used, yield a 100 µl DNA solution, the limit of detection comes close to 5 oocysts/g faeces, which is comparable to the analytical sensitivity reported for a *T. gondii* copro-PCR [44, 45] and a *Besnoitia darlingi* real-time PCR [26]. For comparison, the microscopical evaluation of faecal flotation by sucrose density centrifugation showed a limit of detection of 250 oocysts/g faeces [46].

To demonstrate the practical value of the novel Hham-qPCR1 in examining infections of intermediate hosts, we used tissue samples of GKO mice inoculated with *H. hammondi* oocysts. In contrast to infection with *T. gondii*, *H. hammondi* infections generally become chronic in GKO mice without causing disease (own unpublished data). Even when clinical signs were mentioned in previous studies, they were not described in detail [3, 5]. There are indications, however, that high doses of 10^6^ oocysts cause serious illness in < 10% of inoculated GKO mice [5]. The reason why *H. hammondi* is not as virulent as *T. gondii* seems to be that it replicates only a few times during the tachyzoite stage and eventually differentiates into bradyzoites [34] that no longer cause cell death or harm to the host. As evidenced by laborious and time-consuming histological examinations, cyst-producing (i.e. BAG-1 positive) stages appeared as early as 7 DPI in mice, suggesting that, at least until then, *H. hammondi* multiplied in the tachyzoite stage [3]. Tachyzoite subculture and *in vitro* tissue cyst experiments suggest that there is significant tachyzoite multiplication in *H. hammondi* until about 15–18 DPI [34]. Thereafter, BAG-1-positive tissue cysts were microscopically visible in heart and skeletal muscles and, until 22 DPI, also in mesenteric lymph nodes, liver, lung, kidney and brain. After that time, tissue cysts were microscopically only observed in the lung, heart and skeletal muscles [3]. Our real-time PCR results corroborate these findings. In chronically infected mice (i.e. after 6 weeks of infection), the lowest Ct values were for skeletal muscle, heart and lung tissue (often Ct < 25, and especially in skeletal muscle Ct < 15); however, infections were also frequently observed in the other organs, but high Ct values dominated (often Ct > 30), if these organs were positive at all. This shows that *H. hammondi* can also persist in tissues other than those of the lung, heart or skeletal muscle. The dramatically decreasing Ct values in various skeletal muscles, characterized by ΔCt values of 10.2–13.3 between DPI 6 and DPI 42, are remarkable. They suggest a 10^3^- to 10^4^-fold increase in parasite loads in these tissues during this period of time. In the case of heart tissue, a ΔCt of only 6.5 was observed between DPI 6 and DPI 42, i.e. a 10^2^-fold increase in parasite load. This finding may indicate that striated muscle supports multiplication and persistence of *H. hammondi* better than smooth muscle. This is in contrast to *T. gondii*, for which heart tissue was identified as a site of predilection for chronic *T. gondii* infections in many animal species [47]. As *H. hammondi* and *T. gondii* partially use the same animal species as intermediate hosts, differences in host tissue tropism may prevent, to some extent, interspecies competition in these very closely related parasites.

An increase in the *H. hammondi* parasitic load was also observed in brain tissue, which was characterized by a ΔCt of 11.7, i.e. a 10^4^-fold increase in parasite load, between DPI 6 and DPI 42. However, the parasite load was 10^3^ times lower than in skeletal muscle, which may suggest that *H. hammondi* tachyzoites only reach the brain late during infection. An increase in parasite load may be mainly due to limited tachyzoite and subsequent bradyzoite multiplication in immunologically isolated tissue cysts. This is in accord with earlier observations that there is no cerebral disease in *H. hammondi*-inoculated mice [3].

In mice sacrificed after 42–271 DPI, the results suggested that the parasitic load gradually decreased in all tissues over time. These findings are new, as in the only long-term infection study published so far, a single GKO mouse was used for each day from 22 until 127 DPI, and tissue cysts were observed in the lung and heart and skeletal muscle until 127 DPI, but were not enumerated [3].

Due to the similar, almost identical morphology of tissue cysts of *T. gondii* and *H. hammondi*, chronic *H. hammondi* infections may represent an interesting model for the study of the intra-cyst activity of chemical compounds that might be suitable for the treatment of chronic toxoplasmosis. As the definitive discrimination of *H. hammondi* from *T. gondii* is only possible at the encysted bradyzoite stage, and as re-activation of cysts does not seem to occur in this parasite, *H. hammondi* might be used as a model that could provide information on the true effect of compounds on encysted *H. hammondi* as a proxy for encysted *T. gondii*. The Hham-qPCR1 described here may thus help to quantify the effect of treatments. Moreover, Hham-qPCR1, a novel qPCR, may become a valuable tool for the further elucidation of cell biological differences between the usually pathogenic *T. gondii* and generally non-pathogenic *H. hammondi*, i.e. between two parasites which are genetically very closely related.

## Conclusions

The newly established Hham-qPCR1 assay represents a novel diagnostic tool for epidemiological and cell biological studies on *H. hammondi* in cats as well as in intermediate hosts.

## Supplementary Information

**Additional file 1: Table S1.** Detection of several *Hammondia hammondi* isolates from Austria, the Czech Republic, Denmark, France, Germany and the USA by Hham-qPCR1 [Hham threshold cycle (Ct) value]. The Hham-qPCR1 included an internal control (IC) to assess inhibition.

**Additional file 2: Table S2.***H. hammondi* isolates used to infect gamma interferon gene knockout (GKO) mice [C.129S7(B6)-Ifngtm1Ts/J].

**Additional file 3: Table S3.** Primer and probe sequences tested to establish a *H. hammondi* qPCR targeting the 529-base pair (bp) repeat, HhamREP-529.

**Additional file 4: Table S4.** Summary of the testing of forward and reverse primers and appropriate probes to establish a *H. hammondi* real-time PCR. Two SybrGreen assays per primer pair revealed mean ΔCt values including SD and coefficient of variation (CV) for a borderline *H. hammondi*-positive DNA control and water as a negative control (mean ΔCt_pos-neg_; SD ΔCt_pos-neg_; CV of ΔCt_pos-neg_). For optimal primers (ΔCt_pos-neg_ values of > 4 and a CV < 0.4), all possible TaqMan probes were tested for cross-reactions with *Toxoplasma gondii* (100 ng/µl). All primer combinations that yielded positive PCR reactions (Ct < 40) with *T. gondii* DNA were excluded from further analysis. All TaqMan real-time PCRs with relative fluorescence units (RFUs)  > 1000 were further followed and assessed for the mean Ct value of the borderline *H. hammondi*-positive DNA control. The final primer probe combination used in Hham-qPCR1 consisted of the primers Hham275F and Hham81R and the probe Hham222P (Fig. 2; Additional file 3: Table S3).

**Additional file 5: Table S5**. Hham-qPCR1 results on tissues of 28 GKO mice infected with *H. hammondi* for varying time periods (days post-infection; DPI). For infection, different *H. hammondi* strains and doses were used.

## Data Availability

Data supporting the conclusions of this article are included within the article and its additional files. The raw datasets used and analysed during the present study are available from the corresponding author upon reasonable request.

## Abbreviations

Tg: *Toxoplasma gondii*
Hham: *Hammondia hammondi*
TgREP-529: 529-Base pair repetitive element of *Toxoplasma gondii*
HhamREP-529: 529-Base pair repetitive element of *Hammondia hammondi*
qPCR: Real-time quantitative polymerase chain reaction
ITS: Internal transcribed spacer
Ct: Threshold cycle
GKO: Gamma interferon gene knockout
EGFP: Enhanced green fluorescent protein
IC: Internal control
*SSU-rDNA*: Small subunit ribosomal DNA
DPI: Days post-infection
bp: Base pair

## References

[1] Walzer KA, Adomako-Ankomah Y, Dam RA, Herrmann DC, Schares G, Dubey JP (2013). *Hammondia hammondi*, an avirulent relative of *Toxoplasma gondii*, has functional orthologs of known *T. gondii* virulence genes. Proc Natl Acad Sci USA..

[2] Walzer KA, Wier GM, Dam RA, Srinivasan AR, Borges AL, English ED (2014). *Hammondia hammondi* harbors functional orthologs of the host-modulating effectors GRA15 and ROP16 but is distinguished from *Toxoplasma gondii* by a unique transcriptional profile. Eukaryot Cell..

[3] Dubey JP, Sreekumar C (2003). Redescription of *Hammondia hammondi* and its differentiation from *Toxoplasma gondii*. Int J Parasitol..

[4] Schares G, Vrhovec MG, Pantchev N, Herrmann DC, Conraths FJ (2008). Occurrence of *Toxoplasma gondii* and *Hammondia hammondi* oocysts in the faeces of cats from Germany and other European countries. Vet Parasitol..

[5] Dubey JP, Tilahun G, Boyle JP, Schares G, Verma SK, Ferreira LR (2013). Molecular and biological characterization of first isolates of *Hammondia hammondi* from cats from Ethiopia. J Parasitol..

[6] Schares G, Herrmann DC, Beckert A, Schares S, Hosseininejad M, Pantchev N (2008). Characterization of a repetitive DNA fragment in *Hammondia hammondi* and its utility for the specific differentiation of *H. hammondi* from *Toxoplasma gondii* by PCR. Mol Cell Probes..

[7] Schares G, Ziller M, Herrmann DC, Globokar MV, Pantchev N, Conraths FJ (2016). Seasonality in the proportions of domestic cats shedding *Toxoplasma gondii* or *Hammondia hammondi* oocysts is associated with climatic factors. Int J Parasitol..

[8] Eydelloth M. Experimentelle Untersuchungen ueber das Wirtspektrum von *Hammondia hammondi*. Dissertation, Faculty of Veterinary Medicine, Munich, Germany, 1977.

[9] Mason RW (1978). The detection of *Hammondia hammondi* in Australia and the identification of a free-living intermediate host. Z Parasitenkd..

[10] Frenkel JK, Dubey JP (1975). *Hammondia hammondi*: a new coccidium of cats producing cysts in muscle of other mammals. Science.

[11] Frenkel JK, Dubey JP (1975). *Hammondia hammondi* gen nov., sp. nov., from domestic cats, a new coccidian related to *Toxoplasma* and* Sarcocystis*. Z Parasitenkd..

[12] Christie E, Dubey JP (1977). Cross-immunity between *Hammondia* and *Toxoplasma* infections in mice and hamsters. Infect Immun..

[13] Wallace GD (1975). Observations on a feline coccidium with some characteristics of *Toxoplasma* and *Sarcocystis*. Z Parasitenkd..

[14] Dubey JP (1981). Protective immunity against clinical toxoplasmosis in dairy goats vaccinated with *Hammondia hammondi* and *Hammondia heydorni*. Am J Vet Res..

[15] Dubey JP, Wong M (1978). Experimental *Hammondia hammondi* infection in monkeys. J Parasitol..

[16] Dubey JP, Streitel RH (1976). Further studies on the transmission of *Hammondia hammondi* in cats. J Parasitol..

[17] Burg JL, Grover CM, Pouletty P, Boothroyd JC (1989). Direct and sensitive detection of a pathogenic protozoan, *Toxoplasma gondii*, by polymerase chain reaction. J Clin Microbiol..

[18] Hurtado A, Aduriz G, Moreno B, Barandika J, Garcia-Perez AL (2001). Single tube nested PCR for the detection of *Toxoplasma gondii* in fetal tissues from naturally aborted ewes. Vet Parasitol..

[19] Homan WL, Vercammen M, De Braekeleer J, Verschueren H (2000). Identification of a 200- to 300-fold repetitive 529 bp DNA fragment in *Toxoplasma gondii*, and its use for diagnostic and quantitative PCR. Int J Parasitol..

[20] Reischl U, Bretagne S, Kruger D, Ernault P, Costa JM (2003). Comparison of two DNA targets for the diagnosis of toxoplasmosis by real-time PCR using fluorescence resonance energy transfer hybridization probes. BMC Infect Dis..

[21] Sreekumar C, Vianna MC, Hill DE, Miska KB, Lindquist A, Dubey JP (2005). Differential detection of *Hammondia hammondi* from *Toxoplasma gondii* using polymerase chain reaction. Parasitol Int..

[22] Xia J, Venkat A, Le Roch K, Ay F, Boyle JP (2020). Third generation sequencing revises the molecular karyotype for *Toxoplasma gondii* and identifies emerging copy number variants in sexual recombinants. bioRxiv.

[23] Herrmann DC, Pantchev N, Vrhovec MG, Barutzki D, Wilking H, Fröhlich A (2010). Atypical *Toxoplasma gondii* genotypes identified in oocysts shed by cats in Germany. Int J Parasitol..

[24] Schares G, Pantchev N, Barutzki D, Heydorn AO, Bauer C, Conraths FJ (2005). Oocysts of *Neospora caninum*, *Hammondia heydorni*, *Toxoplasma gondii* and *Hammondia hammondi* in faeces collected from dogs in Germany. Int J Parasitol..

[25] Ho MSY, Barr BC, Marsh AE, Anderson ML, Rowe JD, Tarantal AF (1996). Identification of bovine *Neospora* parasites by PCR amplification and specific small-subunit rRNA sequence probe hybridization. J Clin Microbiol..

[26] Schares G, Dubey JP, Rosenthal B, Tuschy M, Bärwald A, Conraths FJ (2020). Sensitive, quantitative detection of *Besnoitia darlingi* and related parasites in intermediate hosts and to assess felids as definitive hosts for known and as-yet undescribed related parasite species. Int J Parasitol Parasites Wildl..

[27] More G, Schares S, Maksimov A, Conraths FJ, Venturini MC, Schares G (2013). Development of a multiplex real time PCR to differentiate *Sarcocystis* spp. affecting cattle. Vet Parasitol..

[28] Ghosh S, Debnath A, Sil A, De S, Chattopadhyay DJ, Das P (2000). PCR detection of *Giardia lamblia* in stool: targeting intergenic spacer region of multicopy rRNA gene. Mol Cell Probes..

[29] Felleisen RS (1997). Comparative sequence analysis of 5.8S rRNA genes and internal transcribed spacer (ITS) regions of trichomonadid protozoa. Parasitology..

[30] Hoffmann B, Depner K, Schirrmeier H, Beer M (2006). A universal heterologous internal control system for duplex real-time RT-PCR assays used in a detection system for pestiviruses. J Virol Methods..

[31] Talabani H, Asseraf M, Yera H, Delair E, Ancelle T, Thulliez P (2009). Contributions of immunoblotting, real-time PCR, and the Goldmann-Witmer coefficient to diagnosis of atypical toxoplasmic retinochoroiditis. J Clin Microbiol..

[32] Legnani S, Pantchev N, Forlani A, Zini E, Schares G, Balzer J (2016). Emergence of cutaneous neosporosis in a dog receiving immunosuppressive therapy: molecular identification and management. Vet Dermatol..

[33] Galal L, Schares G, Stragier C, Vignoles P, Brouat C, Cuny T (2019). Diversity of *Toxoplasma gondii* strains shaped by commensal communities of small mammals. Int J Parasitol..

[34] Sokol SL, Primack AS, Nair SC, Wong ZS, Tembo M, Verma SK (2018). Dissection of the in vitro developmental program of *Hammondia hammondi* reveals a link between stress sensitivity and life cycle flexibility in *Toxoplasma gondii*. ELife..

[35] Alfonso Y, Fraga J, Gonzalez Z, Jiménez N, Borrero Y, Cox R (2015). Multiplex PCR to detect *T. gondii* infection based on B1 gene and 529 bp repetitive element. J AIDS Clin Res..

[36] Wahab T, Edvinsson B, Palm D, Lindh J (2010). Comparison of the AF146527 and B1 repeated elements, two real-time PCR targets used for detection of *Toxoplasma gondii*. J Clin Microbiol..

[37] Kornacka A, Cybulska A, Moskwa B (2018). Comparison of sensitivity of two primer sets for the detection of *Toxoplasma gondii* DNA in wildlife. Acta Parasitol..

[38] Gomez CA, Sahoo MK, Kahn GY, Zhong L, Montoya JG, Pinsky BA (2019). Dual-target, real-time PCR for the diagnosis of intraocular *Toxoplasma gondii* infections. Br J Ophthalmol..

[39] Belaz S, Gangneux JP, Dupretz P, Guiguen C, Robert-Gangneux F (2015). A 10-year retrospective comparison of two target sequences, REP-529 and B1, for *Toxoplasma gondii* detection by quantitative PCR. J Clin Microbiol..

[40] Veronesi F, Santoro A, Milardi GL, Diaferia M, Branciari R, Miraglia D (2017). Comparison of PCR assays targeting the multi-copy targets B1 gene and 529 bp repetitive element for detection of *Toxoplasma gondii* in swine muscle. Food Microbiol..

[41] Chemoh W, Sawangjaroen N, Nissapatorn V, Sermwittayawong N (2016). Molecular investigation on the occurrence of *Toxoplasma gondii* oocysts in cat feces using TOX-element and ITS-1 region targets. Vet J..

[42] Veronesi F, Santoro A, Milardi GL, Diaferia M, Morganti G, Ranucci D (2017). Detection of *Toxoplasma gondii* in faeces of privately owned cats using two PCR assays targeting the B1 gene and the 529-bp repetitive element. Parasitol Res..

[43] Costa JM, Bretagne S (2012). Variation of B1 gene and AF146527 repeat element copy numbers according to *Toxoplasma gondii* strains assessed using real-time quantitative PCR. J Clin Microbiol..

[44] Salant H, Markovics A, Spira DT, Hamburger J (2007). The development of a molecular approach for coprodiagnosis of *Toxoplasma gondii*. Vet Parasitol..

[45] Salant H, Spira DT, Hamburger J (2010). A comparative analysis of coprologic diagnostic methods for detection of *Toxoplama gondii* in cats. Am J Trop Med Hyg..

[46] Rothe J, McDonald PJ, Johnson AM (1985). Detection of *Toxoplasma* cysts and oocysts in an urban environment in a developed country. Pathology..

[47] Opsteegh M, Maas M, Schares G, van der Giessen J (2016). Relationship between seroprevalence in the main livestock species and presence of *Toxoplasma gondii* in meat (GP/EFSA/BIOHAZ/2013/01). An extensive literature review. Final report..

