# Supplementary material for: A real-time quantitative polymerase chain reaction for the specific detection of Hammondia hammondi and its differentiation from Toxoplasma gondii
Source: Parasit Vectors. 2021 Jan 25;14:78. doi: 10.1186/s13071-020-04571-8 (PMC7830817; doi:10.1186/s13071-020-04571-8)
Supplement: Supplementary file 1 — Additional file 1: Table S1. Detection of several Hammondia hammondi isolates from Austria, the Czech Republic, Denmark, France, Germany and the USA by Hham-qPCR1 [Hham threshold cycle (Ct) value]. The Hham-qPCR1 included an internal control (IC) to assess inhibition. [file 13071_2020_4571_MOESM1_ESM.docx]

**Table S1.**

Detection of several *Hammondia hammondi* isolates from Austria, the Czech Republic, Denmark, France, Germany and the USA by Hham-qPCR1 (Hham Ct value). The Hham-qPCR1 included an internal control (IC) to assess inhibition.

| **DNA ID** | **Oocyst ID** | **Country** | **IC Ct value** | **Hham Ct value** | **Oocysts per ml** | **Log10 (Oocysts per ml)** |
| --- | --- | --- | --- | --- | --- | --- |
| D06633 | VF138094 | France | 31.85 | 23.02 | NA | NA |
| D09478 | VB935409 | Denmark | 31.91 | 21.44 | 3.00 x 10^5^ | 5.48 |
| D09480 | VB943130 | Austria | 32.03 | 26.3 | 7.50 x 10^4^ | 4.88 |
| D09484 | VB944721 | Denmark | 32.47 | 22.57 | 6.75 x 10^4^ | 4.83 |
| D09739 | VB901654 | Denmark | 31.67 | 27.2 | 2.50 x 10^3^ | 3.40 |
| D10148 | VB929869 | Denmark | 32.27 | 19.5 | NA | NA |
| D10151 | VB909755 | Austria | 32.97 | 15.85 | NA | NA |
| D151003 | P15/3653 | Czech Republic | 32.16 | 20.26 | NA | NA |
| D160072 | P16/689 | Germany | 32.78 | 26.06 | 2.65 x 10^5^ | 5.42 |
| D160917 | P16/576 | Germany | N/A | 21.05 | 1.35 x 10^5^ | 5.13 |
| D160953 | P16/2303 | Germany | 32.10 | 27.59 | 4.00 x 10^4^ | 4.60 |
| D170538 | P16/3312 | Germany | 33.02 | 27.19 | 2.65 x 10^5^ | 5.42 |
| D170574 | P17/2213 | Germany | 34.09 | 22.35 | 8.00 x 10^4^ | 4.90 |
| D170575 | P17/2232 | Germany | 32.77 | 23.17 | 5.00 x 10^4^ | 4.70 |
| D170577 | P17/2237 | Germany | 34.06 | 20.14 | 3.70 x 10^5^ | 5.57 |
| D170578 | P17/2238 | Germany | 33.09 | 20.18 | 2.00 x 10^5^ | 5.30 |
| D170579 | P17/2239 | Germany | 33.37 | 20.07 | 5.50 x 10^5^ | 5.74 |
| D180130 | P18/1330 | USA | 35.79 | 19.38 | 5.00 x 10^6^ | 6.70 |
| D180531 | P17/2789 | Germany | 41.16 | 22.03 | 1.56 x 10^7^ | 7.19 |
| D180532 | P17/2842 | Germany | 33.50 | 25.91 | 2.10 x 10^5^ | 5.32 |
| D180533 | P18/1 | Germany | 33.30 | 22.85 | 3.00 x 10^4^ | 4.48 |
| D180534 | P18/375 | Germany | 35.24 | 22.35 | 1.90 x 10^5^ | 5.28 |
| D180535 | P18/433 | Germany | 32.64 | 28.31 | 5.00 x 10^4^ | 4.70 |
| D180572 | P18/2097 | Germany | 35.58 | 22.64 | 4.40 x 10^6^ | 6.64 |
| D190003 | P18/2900 | Germany | 33.35 | 21.35 | 7.00 x 10^5^ | 5.85 |
| D190005 | P18/3689 | Germany | 36.29 | 23.96 | 3.00 x 10^4^ | 4.48 |
| D190926 | P19/2108 | Germany | 32.11 | 21.49 | 9.00 x 10^4^ | 4.95 |
| D200021 | P19/2441 | Germany | 36.03 | 17.48 | 8.00 x 10^4^ | 4.90 |
