# Supplementary material for: A real-time quantitative polymerase chain reaction for the specific detection of Hammondia hammondi and its differentiation from Toxoplasma gondii
Source: Parasit Vectors. 2021 Jan 25;14:78. doi: 10.1186/s13071-020-04571-8 (PMC7830817; doi:10.1186/s13071-020-04571-8)
Supplement: Supplementary file 2 — Additional file 2: Table S2.H. hammondi isolates used to infect gamma interferon gene knockout (GKO) mice [C.129S7(B6)-Ifngtm1Ts/J]. [file 13071_2020_4571_MOESM2_ESM.docx]

**Table S2**

*Hammondia hammondi* isolates used to infect IFN-ɣ knockout (GKO) mice (C.129S7(B6)-Ifngtm1Ts/J)

| **Isolate** | **Duration of the observation period in mice, DPI** | **Country of isolate origin** | **Animal of origin** | **Age of cat, years** | **Gender of cat** | **Type of isolate** |
| --- | --- | --- | --- | --- | --- | --- |
| HH-Iran2010 | 74, 186, 272 | Iran | Cat | NA | NA | Field |
| P15-3653 | 115 | Czech Republic | Cat | NA | NA | Field |
| P17-1842 | 42 | Germany | Cat | 12 | Female | Field |
| P17-2789 | 42 | Germany | Cat | 8 | Female | Field |
| P18-1 | 42 | Germany | Cat | 1 | NA | Field |
| P18-375 | 42 | Germany | Cat | 8 | Male | Field |
| P18-1330 | 3, 6, 42 | USA | Cat | NA | NA | Experimental |
| P18-2097 | 42 | Germany | Cat | NA | NA | Field |
| P18-2102 | 42 | Germany | Cat | 3 | Male | Field |
