# Supplementary material for: A real-time quantitative polymerase chain reaction for the specific detection of Hammondia hammondi and its differentiation from Toxoplasma gondii
Source: Parasit Vectors. 2021 Jan 25;14:78. doi: 10.1186/s13071-020-04571-8 (PMC7830817; doi:10.1186/s13071-020-04571-8)
Supplement: Supplementary file 3 — Additional file 3: Table S3. Primer and probe sequences tested to establish a H. hammondi qPCR targeting the 529-base pair (bp) repeat, HhamREP-529. [file 13071_2020_4571_MOESM3_ESM.docx]

**Table S3.**

Primer and probe sequences tested to establish a *Hammondia hammondi* qPCR targeting a 529 bp repetitive element, HhamREP-529.

| **Orien-tation or probe** | **Name** | **Sequence 5’-3’** | **Calculated melting temperature (Tm)** | **Finally selec-ted** | **Refe-**  **rence** |
| --- | --- | --- | --- | --- | --- |
| Forward | Hham1F | ATC CCA TTC CGG CTT CAG TC | 59.4°C | - | This study |
|  | Hham3F | CCC ATT CCG GCT TCA GTC TT | 59.4°C | - | This study |
|  | Hham17F | AGT CTT TCC ACC CTC CAG GA | 59.4°C | - | This study |
|  | Hham28F | CCT CCA GGA AAC ACA GCC AA | 59.4°C | - | This study |
|  | Hham62F | CTC CCT CTC CGA CTC TCC TC | 63.4°C | - | This study |
|  | Hham44F | CCA AGC CGG AAA CCT CTT CT | 59.4°C | - | This study |
|  | Hham55F | ACC TCT TCT CCC TCT CCG AC | 61.4°C | - | This study |
|  | Hham34F | ATC CCA TTC CGG CTT CAG TCT TTC | 62.7°C | - | Schares et al. (2008)^a^ |
|  | Hham275F | CTA CAA GGG GAG CGT CCT CG | 63.4°C | Yes | This study |
|  | Hham276F | ACT ACA AGG GGA GCG TCC TC | 61.4°C | - | This study |
| Reverse | Hham158R | AGC CGT CTT GGA GGA GAG AA | 59.4°C | - | This study |
|  | Hham157R | GCC GTC TTG GAG GAG AGA AG | 61.4°C | - | This study |
|  | Hham129R | GTA GAT GAA GGC GAG GGT GG | 61.4°C | - | This study |
|  | Hham81R | GAG GAG AGT CGG AGA GGG AG | 63.4°C | Yes | This study |
|  | Hham74R | GTC GGA GAG GGA GAA GAG GT | 61.4°C | - | This study |
|  | Hham63R | AGA AGA GGT TTC CGG CTT GG | 59.4°C | - | This study |
| Probes | Hham75P | GAC TCT CCT CGC TTC CCG ACC | 65.7°C | - | This study |
|  | Hham55P | ACC TCT TCT CCC TCT CCG ACT | 64.0°C | - | This study |
|  | Hham110P | CCA CCC TCG CCT TCA TCT AC | 61.4°C | - | This study |
|  | Hham222P | TCC GGC TTC AGT CTT TCC AC | 59.4°C | Yes | This study |

^a^ Schares G, Herrmann DC, Beckert A, Schares S, Hosseininejad M, Pantchev N, et al. Characterization of a repetitive DNA fragment in *Hammondia hammondi* and its utility for the specific differentiation of *H. hammondi* from *Toxoplasma gondii* by PCR. Mol Cell Probes. 447 2008;22 4:244-51.
